# The generation of chromosomal deletions to provide extensive coverage and subdivision of the *Drosophila melanogaster *genome

**DOI:** 10.1186/gb-2012-13-3-r21

**Published:** 2012-03-22

**Authors:** R Kimberley Cook, Stacey J Christensen, Jennifer A Deal, Rachel A Coburn, Megan E Deal, Jill M Gresens, Thomas C Kaufman, Kevin R Cook

**Affiliations:** 1 Bloomington Drosophila Stock Center, Department of Biology, Indiana University, 1001 E. Third St, Bloomington, IN 47405, USA

## Abstract

**Background:**

Chromosomal deletions are used extensively in *Drosophila melanogaster *genetics research. Deletion mapping is the primary method used for fine-scale gene localization. Effective and efficient deletion mapping requires both extensive genomic coverage and a high density of molecularly defined breakpoints across the genome.

**Results:**

A large-scale resource development project at the Bloomington *Drosophila *Stock Center has improved the choice of deletions beyond that provided by previous projects. FLP-mediated recombination between *FRT*-bearing transposon insertions was used to generate deletions, because it is efficient and provides single-nucleotide resolution in planning deletion screens. The 793 deletions generated pushed coverage of the euchromatic genome to 98.4%. Gaps in coverage contain haplolethal and haplosterile genes, but the sizes of these gaps were minimized by flanking these genes as closely as possible with deletions. In improving coverage, a complete inventory of haplolethal and haplosterile genes was generated and extensive information on other haploinsufficient genes was compiled. To aid mapping experiments, a subset of deletions was organized into a Deficiency Kit to provide maximal coverage efficiently. To improve the resolution of deletion mapping, screens were planned to distribute deletion breakpoints evenly across the genome. The median chromosomal interval between breakpoints now contains only nine genes and 377 intervals contain only single genes.

**Conclusions:**

*Drosophila melanogaster *now has the most extensive genomic deletion coverage and breakpoint subdivision as well as the most comprehensive inventory of haploinsufficient genes of any multicellular organism. The improved selection of chromosomal deletion strains will be useful to nearly all *Drosophila *researchers.

## Background

Chromosomal deletions are important to experimental genetic analysis in two fundamental ways. First, deletions fail to complement loss-of-function mutations in genes located in the chromosomal region of the deletion. This noncomplementation is the basis for using deletions to map mutations to specific chromosomal regions, to screen for new mutations in closely linked sets of genes and to assess the allelic strengths of new mutations. Second, heterozygous deletions can enhance or suppress mutant phenotypes. Reducing the copy number of one gene can modify the phenotype caused by abnormal expression of another gene involved in the same biological process. Modifier screens are a powerful way to identify suites of genes involved in related genetic pathways. *Drosophila melanogaster *tolerates heterozygous deletions of large numbers of genes quite well and its chromosomes are easy to manipulate *in vivo*; consequently, deletions are used more extensively in genetic experiments with *D. melanogaster *than other organisms. Indeed, fine-scale mapping approaches based on meiotic recombination such as SNP mapping are not used widely in fly research, because they are less convenient and precise. Consequently, chromosomal deletion stocks are the most heavily used class of stocks distributed by the Bloomington *Drosophila *Stock Center (BDSC). The demand for a better selection of deletions led us at the BDSC to undertake the large-scale research resource development project described here.

Many methods have been developed for isolating deletions in *Drosophila*. Early methods employed chemical mutagens or irradiation to produce random chromosomal breakpoints. Deletions in specific chromosomal regions were identified by their failure to complement a recessive mutation, by removal of a gain-of-function mutation, or from dominant effects of reduced gene copy number. Obtaining a deletion of a particular size in a desired location was difficult because the mutageneses were inefficient and the positions of breakpoints could not be controlled. Later methods relied on transposons to target deletions to particular chromosomal regions. Deletions flanking *P *and *Minos *elements result from defective transposition events when attempts are made to remobilize the transposons and deletions flanking *Hobo *elements result from recombination between *Hobo *copies following local transpositions [1-6]. While one to two orders of magnitude more efficient than chemical or irradiation screens, transposon remobilization screens still have the disadvantage of breakpoint unpredictability. The positions of breakpoints are typically confined to a small chromosomal region, but they cannot be directed with single-nucleotide certainty. Furthermore, mechanistic constraints make it difficult to obtain large deletions in most remobilization screens.

A more recent method for generating deletions employs FLP recombinase to catalyze recombination between *FRT *sequence elements carried on transposable elements [7]. It is one to two orders of magnitude more efficient than transposon remobilization, the positions of breakpoints can be predicted at the nucleotide level and there are no inherent limitations on deletion size other than aneuploidy effects. This method has been adopted enthusiastically by *Drosophila *geneticists because it has made the isolation of large, multigene deletions and small, single-gene knockouts relatively easy.

There have been three large-scale projects generating deletions using *FLP*-*FRT *technology. The DrosDel Project generated a large collection of insertions of the *FRT*-bearing transposons *P{RS3} *and *P{RS5} *[8] and used them in screens that provided 357 deletion stocks now in the BDSC collection [9]. Exelixis, Inc. generated a larger insertion collection of the *FRT*-bearing constructs *P{XP}*, *PBac{RB} *and *PBac{WH} *[10] and isolated 433 deletions now distributed by the BDSC [5]. These two deletion collections were generated independently and the deletions from these projects being distributed by the BDSC together provide 78% coverage of *Drosophila *euchromatin.

The third deletion project was undertaken at the BDSC and it is the subject of this paper. We began generating deletions before *FLP*-*FRT *technology for deletion screening was generally available. At the time, genomic coverage of approximately 71% was provided by chemical- or irradiation-induced deletions. We were able to provide novel coverage of 5 to 7% of the genome using a *P *element remobilization approach [5], but we retooled our project when the Exelixis *FRT*-bearing insertions were placed into the public domain [10,11]. As we will show, our efforts using the improved technology have resulted in total euchromatic coverage of 98.4%.

To provide such extensive deletion coverage, it was necessary to identify all genes needed in two copies for normal viability and fertility. These haploinsufficient genes are the only barriers to complete deletion coverage in the absence of compensating chromosomal duplications and our work provides the first comprehensive inventory of these genes in *Drosophila melanogaster*. Most remaining gaps in deletion coverage contain a haploinsufficient gene and, because we flanked these genes as closely as possible with pairs of deletions, the gaps are all quite small.

To make it easier for geneticists to take advantage of this broad deletion coverage, the BDSC distributes the 'Deficiency Kit', a selected set of deletions providing maximal genomic coverage with a minimal number of stocks. Here we will describe a new, improved Deficiency Kit composed primarily of molecularly defined deletions from the three deletion projects. It replaces the original Deficiency Kit that consisted of older deletions characterized only at the level of chromosome banding.

While the fraction of the genome covered by deletions in aggregate determines their effectiveness in initially localizing genes, the density of deletion breakpoints determines the resolution of deletion mapping. The more finely the genome is subdivided by breakpoints, the easier it is to map a mutation or genetic modifier to a specific transcription unit. For this reason, we have also worked to improve the distribution of breakpoints across the genome. Because of our efforts, the median interval between breakpoints is now only nine genes and nearly 400 single-gene intervals have been defined.

In brief, we accomplished three goals. First, we improved deletion coverage of the *D. melanogaster *genome substantially and, in the process, provided a complete catalog of haplolethal and haplosterile genes. Second, we updated the Bloomington Deficiency Kit by replacing most chemical- or irradiation-induced deletions with molecularly defined deletions isolated from *FLP*-*FRT *screens. Finally, we improved the resolution of deletion mapping by increasing the density of molecularly defined breakpoints across the genome. The result is a collection of deletions at the BDSC for the rapid and efficient mapping of mutations and modifier loci that is unparalleled among multicellular organisms.

## Results

### Generating molecularly defined deletions by FLP-mediated recombination

**Figure 1:**
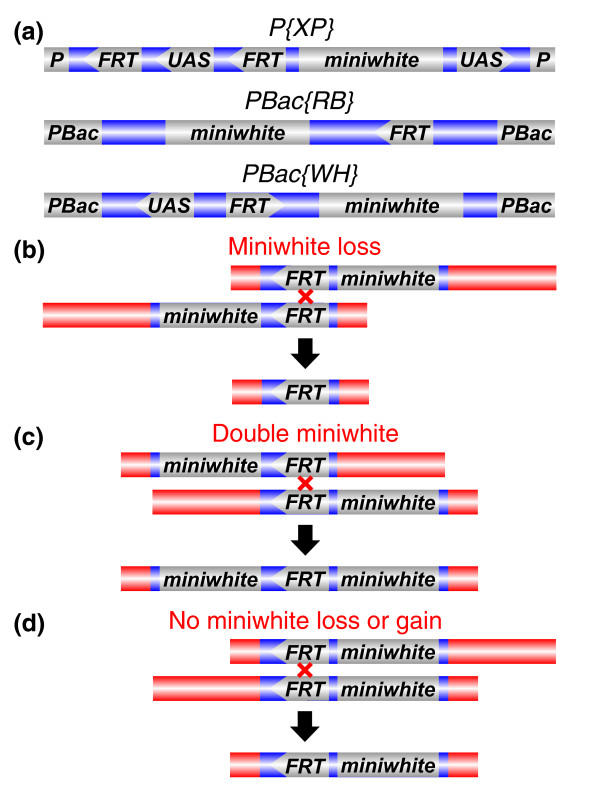
**Generating molecularly defined deletions using Exelixis *FRT*-bearing transposon insertions**. **(a) **The structure of the *P{XP}*, *PBac{RB}*and *PBac{WH} *constructs. The *miniwhite*-marked *P *element or *piggyBac *(*PBac*) constructs carry one or two *FRT *sequences with the indicated orientations. (*miniwhite *is a version of the *white *gene engineered for compact size.) *P{XP} *and *PBac{WH} *constructs also carry *UAS *sequences oriented to allow GAL4-induced expression of genes near the genomic insertion sites of the constructs. One *UAS *sequence in *P{XP} *can be removed by FLP-mediated recombination and it is likely that this cassette is absent from most deletion chromosomes, though we did not assay for it in our deletion chromosomes. **(b-d) **Simplified diagrams of FLP-mediated recombination events generating deletion chromosomes with different *miniwhite *copy numbers. (b) Our most frequently used screening strategy, where deletions are identified based on loss of *miniwhite *and the resulting white eye color. (c) The alternative strategy of identifying deletions based on increased *miniwhite *copy number relative to progenitor chromosomes and the resulting darker eye color. (d) Deletions can be recovered without a decrease or increase in *miniwhite *copy number, though we did not undertake such screens.

The system for generating deletions devised at Exelixis involves the use of three *FRT*-bearing transposon constructs (Figure 1a) [5,10]. Each construct carries the *miniwhite *marker and at least one *FRT *sequence. Deletions can be recovered by combining pairs of insertions *in trans *in the presence of an inducible genomic source of FLP recombinase. FLP-mediated recombination generates a deletion if the *FRT *sequences are arranged in the same relative orientation. If the *miniwhite *markers lie internal to the *FRT *sequences, deletion-bearing progeny will have white eyes from elimination of both copies of *miniwhite *upon recombination (Figure 1b). If they lie external to the *FRT*s, deletion-bearing progeny often have darker eyes than flies in either single-*FRT *progenitor strain from the presence of two copies of *miniwhite *(Figure 1c). We generated 87% of our deletions using construct combinations eliminating *miniwhite *markers; the remaining deletions were isolated in progeny bearing two copies of *miniwhite*. Pairs of insertions can also be used to recover deletions with no net gain or loss of *miniwhite *markers (Figure 1d), but such screens were not attempted in this project.

Approximately 16,500 *FRT*-bearing transposon insertions on the *X *chromosome and autosomes were generated and placed into public distribution by Exelixis [10,11]. The density of insertions afforded by such a large collection gave us flexibility in designing deletions. As we will describe below, convenient insertions were usually available so that we could flank haploinsufficient genes closely with pairs of deletions in our efforts to maximize deletion coverage and improve the distribution of breakpoints. In some chromosomal regions, however, the scarcity of insertions limited our efforts.

We isolated 793 deletions. They are described in detail in Additional file 1 and their breakpoint positions are shown relative to annotated genes in Additional file 2. Because we were primarily concerned with breakpoint placement, we did not standardize the size of the deletions. We did not generate deletions with heterochromatic breakpoints because most heterochromatic *P{XP}*, *PBac{WH} *and *PBac{RB} *insertions have not been localized unambiguously. In the screens that were successful, one deletion was recovered for every approximately 50 progeny screened with a range of roughly 1 in 10 to 1 in 1,000. We retained only one deletion from each screen for the BDSC collection.

We confirmed the existence of deletions by demonstrating that genes located between the progenitor insertions were missing. We usually designed deletions so they encompassed at least one gene with an existing loss-of-function mutation to use in complementation tests. Only two deletions designed early in the project were small enough to avoid removing vital genes. We also used preexisting deletions in complementation tests with new deletions if the progenitor *FRT*-bearing insertions were not themselves homozygous lethal to demonstrate that at least one vital gene was deleted. If an appropriate complementation test was not available, we verified the presence of a deletion by polytene cytology or by demonstrating that a particular internal DNA fragment could not be PCR amplified from the deletion chromosome. Eighteen new deletions showed dominant phenotypes expected for deleting known haploinsufficient genes (Additional file 1).

We also verified that the expected deletion event occurred using the 'hybrid PCR' method [5]. In brief, PCR primers within each *FRT*-bearing progenitor insertion can amplify a fragment only if they are juxtaposed by FLP-mediated recombination. We tested 93% of the deletions lacking *miniwhite *markers by this method and all gave the expected result (Additional file 1). We used a positive hybrid PCR test as the sole criterion for verifying only six deletions; all other deletions were also confirmed on the basis of deleted genes or sequences.

The Exelixis insertions were generated in a uniform genetic background [10] and we maintained this background in most deletion screens as described in Parks *et al.*[5]. Noninsertion chromosomes specific to our screens were substituted into the standard background prior to use. Minimizing genetic variability in the deletion stocks makes them more valuable in the analysis of background-sensitive phenotypes such as behavioral traits. It was necessary to establish 27 deletions in stock with nonstandard balancers due to dominant effects of the deletions on viability or fertility or due to noncomplementation with mutations on standard balancers (Additional file 1).

Deletion stocks were placed into public distribution as soon as the deletions were verified; consequently, many deletions have been in widespread use for years and have contributed to published research (for examples, [12-18]). Details of each deletion screen have been documented in FlyBase and references to the individual reports are given in Additional file 1. FlyBase developed a graphical interface for viewing molecularly defined deletions during our project [19] and the BDSC website lists available deletions [20].

We conducted 708 screens that produced no deletions and we were often able to determine the cause of the failure. First, the rate of recovering deletions was often low. We were able to generate most deletions with modest-sized screens, but some deletions required larger screens. Four percent of deletions were recovered only after additional rounds of screening. Five percent of the screens failed even though both progenitor insertions had been used in successful screens, there was no haploinsufficient gene in the region and the deletion would not have been particularly large, suggesting small screen size as the most likely explanation for failure. Second, the insertions in many stocks did not map as described. We saw evidence that insertions were mismapped in 12% of the unsuccessful screens. (All eight of the fourth chromosome deletion screens were in this category, suggesting that a high proportion of the fourth chromosome insertion stocks are not as represented. We abandoned plans for additional screens without validating the remaining putative fourth chromosome insertion stocks.) Third, in 6% of the failed screens, putative deletions could not be established in stock due to lethality and/or sterility, even though no haploinsufficient genes existed in the region. We suspect that the abnormal juxtaposition of genic regions upon deletion results in deleterious neomorphic phenotypes more often than is generally appreciated. Fourth, 5% of unsuccessful screens failed because flies in the intermediate crosses were weak, sterile or dead. We attribute these problems to noncomplementation and dominant interactions between mutations in the stocks. Fifth, 10% of unsuccessful screens failed because we intentionally deleted haploinsufficient genes. (We will discuss these experiments below.) The remaining screens probably failed from small screen sizes or mismapped insertions. They all involved at least one insertion stock that had not been used successfully in another screen. From these experiences, we recommend that anyone planning *FLP*-*FRT *screens verify the positions of progenitor insertions, plan screens larger than our routine screen sizes and, if possible, attempt to create deletions of a region using more than one combination of progenitor insertions.

False positives were recovered in many screens. For example, white-eyed progeny carrying no deletion were recovered in screens designed to eliminate the *miniwhite *markers from both progenitor insertions upon FLP-mediated recombination (Figure 1b). While false positives did not prevent the recovery of deletions, they increased the workload in the verification step. At least 9% of screens successfully producing deletions also produced false positives. Inexplicably, *X *chromosome screens were much more likely to give false positives than autosomal screens (49% versus 4%). At least 23% of the screens producing no deletions gave false positives, again, with a disproportionate number involving the *X *chromosome rather than an autosome (56% versus 15%). False positives were also seen in Exelixis and DrosDel screens (A Parks, personal communication) [9]. Consequently, we recommend testing multiple putative deletions in all screens. Though the false positives have not been characterized in detail, we surmise they originate from the repair of DNA damage associated with abnormal FLP-mediated recombination events.

### Improving deletion coverage of the genome

The first goal of our project was to improve deletion coverage of the genome. As summarized in Table 1 and shown in detail in Additional file 2, 98.4% coverage of euchromatic genes is now available using deletions distributed by the BDSC regardless of their origin. The deletions we generated (the 'BSC' deletions) encompass 81.5% of euchromatic genes as compared to 65.2% for the DrosDel deletions at Bloomington and 47.4% for the Exelixis deletions. Together, the molecularly defined deletions from the three large-scale projects provide 94.9% coverage with 17.9% of euchromatic genes removed exclusively by BSC deletions. The remaining genomic coverage is provided by chemical- or irradiation-induced deletions (3.4%) and *FRT*-derived deletions from individual investigators (0.1%).

**Table 1:** Percent coverage of euchromatic genes by deletions

Chromosome arm	BSC deletions	Exelixis deletions^a^	DrosDel deletions^a^	BSC, Exelixis and DrosDel deletions	Unique to BSC deletions^b^	Other deletions^c^	All available deletions
*X*	82.7	18.0	57.6	92.3	26.9	5.9	98.1
*2L*	70.5	68.2	71.7	96.0	12.0	3.0	98.9
*2R*	88.5	45.7	52.4	95.9	26.8	2.3	98.2
*3L*	84.1	39.3	69.4	95.3	15.3	2.5	97.5
*3R*	83.0	59.6	73.0	95.7	11.4	3.2	98.9
*4*	0	0	54.1	54.1	0	42.4	96.5
Total	81.5	47.4	65.2	94.9	17.9	3.5	98.4

^a^Coverage by Exelixis or DrosDel deletion stocks maintained at the BDSC. Some deletions reported by Exelixis [5] were false positives; stocks for other deletions were lost. Stocks for some DrosDel deletions [9] were too weak to maintain. ^b^Coverage provided by BSC deletions, but not Exelixis or DrosDel deletions. ^c^Chemical- or irradiation-induced deletions plus *FRT*-derived deletions from individual investigators.

The single biological impediment to complete deletion coverage is the existence of genes that must be present in more than one copy for viability and fertility. Deletions of these haplolethal and haplosterile genes cannot be recovered in the absence of a compensating chromosomal duplication. (Obviously, haploinsufficiency pertains to *X*-linked genes only in females where two gene copies are normally present.) Duplications had been isolated for some haplolethal and haplosterile genes before we began our work, but generating new duplications in order to screen for deletions was beyond the scope of this project. Instead, we generated pairs of deletions to flank haplolethal and haplosterile genes as closely as possible as a way to maximize deletion coverage. This strategy allowed us to improve coverage substantially, but it precluded 100% coverage. The sizes of gaps in coverage were determined by the availability of *FRT*-bearing transgene insertions; consequently, some genes closely linked to haplolethal and haplosterile genes also remain undeleted. We chose not to use preexisting duplications to screen for deletions of haploinsufficient genes because we wanted to preserve a consistent genetic background in our deletion stocks. We were also aware of the fact that deletions of haploinsufficient genes have limited experimental value: the phenotypes of haploinsufficient genes often confound the interpretation of complementation tests and they enhance so many other mutant phenotypes that they are generally viewed as nuisances in modifier screens.

If we consider the distribution of deletion breakpoints for the DrosDel, Exelixis and BSC deletions, we see there is a total of 81 gaps in deletion coverage across the entire euchromatic genome (Additional file 2). Fifty-five gaps contain haploinsufficient genes, five gaps (including three with haploinsufficient genes) lie adjacent to centric heterochromatin where screening was not possible, three gaps (including one with a haploinsufficient and one adjacent centric heterochromatin) lie on the fourth chromosome where we recovered no deletions and 23 gaps are not covered despite the absence of haploinsufficient genes. The median size of the gaps is only 7 genes and the gaps range in size from 1 to 36 genes (1 to 27 if a large fourth chromosome gap is disregarded). We made multiple attempts to provide deletion coverage for most gaps lacking haplolethal or haplosterile genes, but we were unsuccessful for the reasons outlined above.

Fortunately, many gaps in coverage between BSC, Exelixis and DrosDel deletions are covered by older deletions or *FRT*-derived deletions from individual investigators. We have no clear explanation for the failure of *FLP*-*FRT *screens in regions where older deletions provided coverage. Although the haplosterility or haplolethality of some genes might depend on genetic background, it is unlikely this is the case for a large number of genes. Screens with different progenitor insertions to the ones we used might prove successful. When deletions from all sources are considered - including those maintained in stock with a duplication - only 35 gaps in coverage remain. {AU comment: It seems to me that em dashes should be used here rather than hyphens.}Thirty-two gaps contain haploinsufficient genes, three gaps (including one with a haploinsufficient gene) lie adjacent to centric heterochromatin, and two gaps (including one adjacent to centric heterochromatin) lie on the fourth chromosome. The median gap size is 7 genes with a range of 1 to 18 genes. We verified that the older deletions span gaps with complementation tests and PCR amplifications. In many cases, these verification experiments also allowed us to map deletion breakpoints of the older deletions more precisely (Additional file 2). In addition, the breakpoints of three gap-covering deletions (*Df(2L)C144*, *Df(2R)CX1 *and *Df(3L)ZN47*) were mapped with comparative genomic hybridization microarrays at the same time breakpoints of *X *chromosome duplications from another BDSC resource project were localized [21].

### Identifying haploinsufficient genes

Although it was necessary to identify haplolethal and haplosterile genes in order to improve deletion coverage, most were poorly mapped and few had been associated with annotated genes when we began our project. Consequently, the process of planning deletion screens was intertwined with the task of identifying haploinsufficient genes. When there were no hints about the molecular identity of a haploinsufficient gene, we faced the tedious process of iterative deletion screening to narrow its location.

Fortunately, most haploinsufficient genes in *Drosophila *encode protein components of cytoplasmic ribosomes (*Rp *genes) or translation initiation factors (*eIF *genes) and are associated with the Minute syndrome, a characteristic set of phenotypes including short, thin bristles and slower development [22]. All *Minute *genes are associated with some degree of haplolethality and haplosterility, but the phenotypes of many *Minute *genes are not severe enough to prevent the recovery of deletions in the absence of duplications. We assured that all *Minute *genes were flanked as closely as possible with deletions and we attempted to delete 34 *Minute *genes to assess their viability and fertility. Deletions for 7 *Minute *genes could be established in simple heterozygous stocks, but deletions for the remaining 27 could not be recovered or heterozygous stocks were too weak to maintain long term. We did not attempt to delete *Minute *genes where evidence already existed for strong lethality or sterility effects. Table 2 shows *Minute *genes with viability or fertility effects strong enough to prevent the recovery of deletions in the absence of a duplication; Table 3 shows *Minute *genes with weaker effects. Because *Minute *phenotypes lie on a continuum and vary by genetic background, genes with intermediate effects might be classified differently in other experiments. Additional files 2 and 3 provide details of deletion coverage near *Minute *genes. This inventory updates the number of *Minute *loci given in Marygold *et al.*[22] from 65 to 66 with the reclassification of *RpL23*.

**Table 2:** Haplolethal and haplosterile genes

Gene	Location
*X *chromosome	
*RpL36*	1B12
*RpL35*	5A11
*RpL17*	6C10
*RpS6*	7C2
*Fs(1)10A *region	10A4-10
*Hdl *region	12A7-9
*RpL37a*	13B1
*RpS19a*	14F4
*RpS5a*	15E5-7
*wupA *region	16F7
*RpS10b*	18D3
Chromosome arm *2L*	
*dpp*	22F1-3
*RpL37A*	25C4
*RpL36A*	28D3
*RpS13*	29B2
*RpL13*	30F3
*RpL7*	31B1
*RpL9*	32C1
*RpL24*	34B10
Chromosome arm *2R*	
*RpL31*	45F5
*RpS11*	48E8-9
*RpS15*	53C8
*RpL18A*	54C3
*RpL11*	56D7
*RpL23*	59B3
*RpL12*^a^	60B7
*RpL39*^a^	60B7
Chromosome arm *3L*	
*RpL23A*	62A10
*RpL8*	62E7
*RpL28*	63B14
*RpL18*	65E9
*RpL14*	66D8
Haplolethal region	67D10-E1
*RpS4*	69F6
*RpL10*	80A
Chromosome arm *3R*	
*RpL35A*	83A4
*RpL13A*	83B6-7
*Tpl *region	83E1-2
*RpL34b*	85D15
*Ms(3)88C *region	88C9-10
*Su(var)3-9*	88E8
*Abd-B*	89E4-5
*RpS3*	94E13
*RpL27*	96E9-10
*RpL4*	98B6
*RpS8*	99C4
*RpL32*	99D3
*RpS7*	99E2
*RpL6*	100C7

^a^A Minute phenotype results from deleting chromosomal region 60B, but it may be associated with haploinsufficiency of *RpL12*, *RpL39 *or both genes.

**Table 3:** *Minute *genes not associated with strong viability and fertility effects

Gene	Location
*X *chromosome	
*RpL7A*	6B1
*RpS28b*	8E7
*RpS15Aa*	11E11-12
*eIF-2alpha*	14C6
Chromosome arm *2L*	
*RpLP1*	21C2
*RpS21*	23B6
*RpL40*	24E1
*RpL27A*	24F3
*RpS27A*	31E1
*RpS26*	36F4
*RpL21*	40A-B
*RpL5*	40B
Chromosome arm *2R*	
*RpL38*	41C-E
*RpS23*	50E4
*RpLP2*	53C9
*RpS18*	56F11
*RpS16*	58F1
*RpS24*	58F3
*RpL19*	60E11
Chromosome arm *3L*	
*RpS17*	67B5
*RpS9*	67B11
*RpL15*	80F
Chromosome arm *3R*	
*RpS29*	85E8
*RpS25*	86D8
*RpS20*^a^	93A1
*RpS30*^a^	93A2
*RpS27*	96C8
Chromosome *4*	
*RpS3A*	101F1

^a^A Minute phenotype results from deleting chromosomal region 93A, but it may be associated with haploinsufficiency of *RpS20*, *RpS30 *or both genes.

Nine haplolethal or haplosterile loci do not encode ribosome-associated proteins (Table 2; Additional files 2 and 3). Four of the non-*Minute *genes (*Fs(1)10A*, *Hdl*, the unnamed haplolethal in 67DE and *Ms(3)88C*) have not been mapped to single transcription units, but the DrosDel Project localized *Ms(3)88C *to a small region by successive rounds of screens [9] and we localized *Fs(1)10A*, *Hdl *and the 67DE haplolethal similarly. The *Tpl *locus corresponds to a repetitive gene cluster [23]. We defined a small region within the *Tpl *cluster that could not be further narrowed by deleting from the proximal and distal sides using the available *FRT *insertions, but it is possible that viability simply requires a minimal number of gene repeats. Thirty-five of our failed screens were devoted to mapping these non-*Minute *haploinsufficient loci.

Tables 2 and 3 present comprehensive inventories of haplolethal, haplosterile and *Minute *genes in *D. melanogaster*, but we also compiled a list of haploinsufficient genes associated with developmental or cellular phenotypes that do not affect viability or fertility enough to prevent the recovery of deletions (Table 4; Additional file 2). All were removed by molecularly defined deletions, but we made no attempt to flank these genes closely with deletions. Only the *Hup *locus has not been mapped to a transcription unit. We did not identify new haploinsufficient genes with obvious external morphological phenotypes and few are likely to exist. Additional haploinsufficient genes with cellular phenotypes may have been described in the literature and others will probably be identified in the future, so Table 4 is undoubtedly incomplete. We do not know how many haploinsufficient genes might exist with moderate viability or fertility effects similar to the *Minute *genes in Table 3 yet have no obvious external morphological effects.

**Table 4:** Haploinsufficient loci with developmental or cellular phenotypes not associated with strong haplolethality or haplosterility

Gene	Location	Phenotype	Reference^a^
*N*	3C7-9	Wing notching	[47]
*Hup*^b^	7BC	Pronotal outgrowth	[48]
*run*	19E2	Segmentation defects	[49]
*S*	21E4	Eye roughness	[50]
*Pkd2*	33E3	Reduced smooth muscle contractility	[51]
*b*	34D1	Darker body color	[52]
*Mhc*	36B1	Muscle defects	[53]
*lok*	38B2	No apoptosis after telomere loss	[54]
*vg*	49E1	Wing notching	[55]
*Np/CG34350*^c^	45A1	Notopleural bristle length	[56]
*Pcl*	55B8	2nd to 1st leg transformation	[57]
*bs*^d^	60C6	Wing venation defects	[58]
*Dll*	60E2	Antenna to leg transformation	[59]
*Kr*	60F5	Segmentation defects	[60]
*mtrm*	66C11	Increased female nondisjunction	[61]
*Pc*	78C6-7	2nd to 1st leg transformation	[62]
*Scr*	84A5	1st to 2nd leg transformation	[63]
*Tm2*	88E13	Muscle defects	[64]
*Act88F*	88F5	Muscle defects	[65]
*Ubx*	89D9	Haltere to wing transformation	[66]
*Dl*	92A1-2	Wing venation defects	[67]
*bnl*	92B2-3	Abnormal tracheal branching	[68]
*H*	92F3	Bristle shaft to socket transformation	[33]
*e*	93C7-D1	Darker body color	[48]
*p53*	94D10	No apoptosis after telomere loss	[54]
*Mlc2*	99E1	Muscle defects	[69]

^a^Reference originally describing haploinsufficiency. ^b^*Humeral patch *(*Hup*) is the only locus in this list not mapped to a specific transcription unit. ^c^Allelism of *Notopleural *(*Np*) and *CG34350 *demonstrated by Laurence von Kalm (personal communication). ^d^*Plexate *(*Px*) is a haploinsufficient locus closely linked or allelic to *blistered *(*bs*) with similar phenotypes. We have assumed allelism.

The non-*Minute *haploinsufficient genes listed in Tables 2 and 4 implicate no single biological pathway or process, though there are a few discernable themes. The largest subset encodes the muscle components actin (*Act88F*), myosin (*Mhc *and *Mlc2*) and tropomyosin (*Tm2*) as well as a group of closely linked, muscle-related genes regulated by a haplolethal sequence within an intron of the *Troponin I *(*wupA*) gene [24]. This subset may also include *Hdl*, which may correspond to *Troponin T *(*up*) [21,25]. Like the ribosomal protein genes, these genes may be particularly dosage sensitive because muscle assembly requires minimal levels or a particular stoichiometry of component proteins [22,24,26]. Other definable subsets encode homeodomain proteins (*Abd-B*, *Dll*, *Scr *and *Ubx*), *Notch *pathway components (*Dl*, *H *and *N*), *Polycomb *group repressor proteins (*Pc *and *Pcl*), apoptosis regulators (*lok *and *p53*) and melanin biosynthetic enzymes (*b *and *e*).

### Assembling a new Deficiency Kit

For years, the BDSC has maintained and distributed a set of deletions providing maximal genomic coverage using the fewest deletions deemed practical. The 'Deficiency Kit' was established before *FLP*-*FRT *deletions existed and deletions were added to the original deletion set when they provided novel coverage. Consequently, the kit consisted mostly of deletions with breakpoints mapped only at the level of polytene cytology. With large collections of molecularly defined deletions available, the kit had become outdated.

As the second goal of our project, we created a new Deficiency Kit and began distributing it in July 2009 while we were still screening for new deletions. We chose a tiling path of *FRT*-derived deletions from the three deletion projects and from individual investigators to provide maximal coverage. The remaining gaps in coverage were filled with older deletions where possible. In many cases, the older deletions were maintained in stock with duplications, allowing us to delete many regions containing haploinsufficient loci. To create a kit better suited for genetic modifier screens, we included the deletion pairs most closely flanking haploinsufficient genes even when they were removed completely by older deletions. Older deletions also allowed us to cover heterochromatic regions where *FLP*-*FRT *deletions currently do not exist and the fourth chromosome where few molecularly defined deletions exist. As we generated new deletions, we updated and improved the kit. Table 5 shows a summary of genomic coverage provided by the current kit. Additional file 4 lists the contents of the Deficiency Kit and Additional file 2 shows the deletion breakpoints relative to annotated genes. Information on the Deficiency Kit is also provided on the BDSC website [27].

**Table 5:** The Bloomington Deficiency Kit

Chromosome	Percent coverage of euchromatic genes	Number of stocks
*X*	98.1	92
*2L*	98.9	100
*2R*	98.2	90
*3L*	97.5	76
*3R*	98.9	104
*4*	96.5	7
Total	98.4	468^a^

^a^One stock carries deletions in both 3L and 3R.

Because the average size of the DrosDel, Exelixis and BSC deletions is smaller than the average size of the deletions in the previous Deficiency Kit, the new kit is larger (468 versus 271 deletions). Nevertheless, the advantages of molecularly mapped breakpoints and consistent genetic backgrounds offset the inconvenience of handling more stocks in experiments. As we will show in the next section, once a phenotype is mapped to a chromosomal interval using the new Deficiency Kit, its position can be narrowed to a very small interval or even a single transcription unit using additional molecularly defined deletions.

### Extensive subdivision of the genome with deletion breakpoints

When a gene is mapped with deletions, it is usually localized to a chromosomal interval defined by adjacent breakpoints of different deletions rather than the breakpoints of a single deletion. For example, a mutation would map between the distal breakpoints of two overlapping deletions if it complemented the proximal deletion but not the distal deletion. Consequently, the critical factor determining the resolution of deletion mapping is the density of deletion breakpoints rather than deletion size.

**Figure 2:**
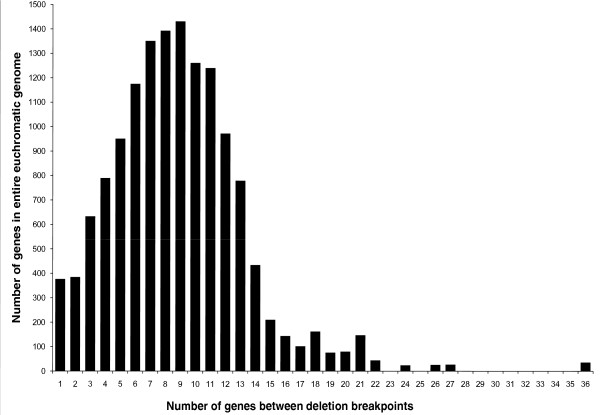
**Frequency distribution of the number of genes between molecularly defined deletion breakpoints**. The number of genes in a chromosomal interval between adjacent deletion breakpoints is shown on the x-axis and the number of intervals in the *Drosophila *genome with those sizes is shown on the y-axis. Because BSC, Exelixis and DrosDel deletions overlap extensively, the intervals were usually defined by breakpoints of different deletions. The median interval size is only nine genes. For simplicity, the *Stellate *gene cluster in chromosomal region 12E, the *histone *gene cluster in 39D and the *5S rRNA *gene cluster in 56E were counted as single genes.

The third goal of our project was to improve the distribution of deletion breakpoints across the genome and our target was to generate deletions so that the intervals between adjacent breakpoints would contain no more than a dozen protein-coding genes. We examined the distribution of breakpoints provided by the DrosDel and Exelixis deletions and undertook screens to interpose new breakpoints between them (Additional file 2). Figure 2 shows the distribution of interval sizes provided by deletions from the three projects. To construct this distribution, we assigned every euchromatic gene a position between adjacent deletion breakpoints based on where it would map uniquely if a mutation in the gene were complementation tested with the deletions. These hypothetical complementation tests allowed us to assign a gene to a single interval even when a deletion breakpoint fell within the gene. The median interval size for the entire genome is nine genes. The median interval size for chromosome arms *X*, *2R*, *3L *and *3R *is also nine genes, but the median size for *2L*, where there are more *FRT*-derived deletions, is only seven genes.

Many intervals exceeded our target size of 12 genes, though the largest interval contains only 36 genes (it lies on the fourth chromosome, where our screens were unsuccessful; the largest interval elsewhere contains 27 genes). The largest intervals could not be subdivided because no *FRT*-bearing transposon insertions existed in the interval or repeated attempts to subdivide the region with existing insertions failed. Other intervals exceeded our target size because gene annotations changed in the course of the project or because we counted genes for noncoding RNAs such as microRNAs and small nucleolar RNAs, which we did not consider in planning the deletion screens due to their poor annotation at the time.

As Figure 2 shows, it is now possible to map most mutations and genetic modifiers to very small groups of candidate transcription units with simple crosses. The deletion breakpoints even define 377 single-gene intervals. This degree of genomic subdivision makes deletion mapping the method of choice for most gene localization experiments in *D. melanogaster*.

## Discussion

The enormous benefit of using FLP-mediated recombination between *FRT*-bearing transposon insertions to generate chromosomal deletions is absolute predictability. Deletion breakpoints can be positioned with single-nucleotide certainty. Homologous recombination methods [28] are the only other approaches in *D. melanogaster *allowing one to plan deletion screens with such precision. The advent of these molecularly defined deletions fundamentally changed the experience of deletion mapping. Previously, the breakpoints of most deletions had been mapped only at the resolution of polytene chromosome analysis, which, even at its best, positioned breakpoints in relatively large chromosomal intervals. The uncertainty of breakpoint positions in older deletions made high-resolution mapping difficult. In contrast, deletion mapping with *FLP*-*FRT *deletions is straightforward and unambiguous. The obvious advantages of these deletions led to their immediate and enthusiastic adoption by *Drosophila *geneticists. While the older deletions are still valuable, they are not used as frequently as they once were. Approximately 85% of deletion stocks currently ordered from the BDSC are *FRT*-derived deletions.

Our project at the BDSC was one of three large-scale projects generating *FLP*-*FRT *deletions. The Exelixis and DrosDel Projects also generated extensive genomic coverage, but our project is distinguished by its emphasis on improving deletion coverage by minimizing undeleted regions containing haploinsufficient genes and by its emphasis on genomic subdivision. Haplolethal and haplosterile loci were not a big consideration in the planning of the Exelixis deletions while the relatively low density of the DrosDel *FRT*-bearing transposons across the genome did not, on average, allow the DrosDel deletions to confine haploinsufficient genes to small undeleted intervals. Our project stressed flanking haploinsufficient genes as closely as possible with pairs of deletions as the most practical approach to improving deletion coverage even before we started using the *FLP*-*FRT *system. The deletions we generated by the hybrid element insertion method [5] were also designed to provide novel coverage. Our attention to haploinsufficient genes is largely responsible for improving overall genome coverage beyond that provided by either the older deletions or the DrosDel and Exelixis collections. It has also helped produce the most comprehensive inventory of haploinsufficient genes to date in *Drosophila *or any other multicellular eukaryote.

The first chromosomal deletion was discovered in *D. melanogaster *[29] and *Drosophila *now boasts the best genomic deletion coverage and subdivision of any animal. Total coverage is even roughly equivalent to that in the yeasts *Saccharomyces cerevisiae *and *Schizosaccaromyces pombe*, where high-throughput homologous recombination approaches are available [30,31]. Despite this accomplishment, our approach of flanking haploinsufficient genes as closely as possible with deletions, rather than first producing new duplications and subsequently using them in deletion screens, precluded complete genomic coverage. The recent generation of *X *and second chromosome duplications [9,21] and the development of *ΦC31 *transformation technology for the creation of new multigene duplications [25,32] will make it much easier to isolate deletions for chromosomal regions containing haploinsufficient genes. Further improvements in deletion coverage will doubtlessly occur as these resources and techniques are exploited.

It is remarkable how few haploinsufficient genes are present in the *D. melanogaster *genome - particularly when one considers that the vast majority fall into a single class, the *Minute *genes, and produce a consistent set of phenotypes. {AU comment: It seems to me than an em dash should be used rather than a hyphen.} There are very few haplolethal or haplosterile genes of any kind. In fact, most deletions in *Drosophila *appear to have no dominant phenotypic effect and, on average, one-twentieth of a chromosome arm must be deleted before significant aneuploidy-associated phenotypes are seen [33,34]. This stands in stark contrast to humans, where haploinsufficiency causes hundreds of genetic diseases [35-38]. Even though the *Drosophila *genome has never been systematically surveyed for weak fitness effects arising from reduced gene dosage and one could argue that subtle mutant phenotypes are more easily recognized in humans, the tolerance *Drosophila *shows to hypoploidy must reflect fundamental differences in the abilities of organisms to compensate for changes in gene dosage.

The extensive genomic coverage and subdivision provided by molecularly defined deletions in *Drosophila *presents researchers with the ability to map mutations and genetic modifiers to very small chromosomal intervals with a few rounds of simple crosses. While it is sometimes necessary to generate additional deletions for further localization, it is often possible to identify an obvious candidate based on its sequence characteristics or previously characterized mutant phenotypes. Gene identification is much easier and more efficient than it was even a decade ago.

The mapping of *X*-linked mutations and modifiers benefits from the *X *chromosome deletions described here as well as duplications of *X *chromosomes that were generated at the BDSC in a concurrent project [21]. We have now isolated *Y*-linked duplications for 97% of the *X *chromosome (data not shown). These duplications can be paired with *X *chromosome deletions to allow inheritance of the deletions from fathers (indeed many stocks with deletion-duplication pairs are already distributed by the BDSC) and they can be used to map mutations by phenotypic rescue and identify hyperploid modifiers. Using the molecularly defined *X *chromosome deletions, the *Y*-linked *X *chromosome duplications and interchromosomal duplications of *X *segments generated by *ΦC31 *transformation [25], phenotypes can be mapped with near single-gene resolution.

## Conclusions

We have presented the results of a large-scale resource development project at the BDSC. We substantially improved the overall genomic coverage provided by chromosomal deletions as well as the density of deletion breakpoints. Our efforts will simplify experiments utilizing deletions to map mutations or identify dosage-sensitive enhancers and suppressors of mutant phenotypes. To aid researchers in conducting such experiments efficiently, we organized deletions providing maximal genomic coverage into a Deficiency Kit. We also made a complete inventory of haplolethal and haplosterile genes and compiled information about other haploinsufficient genes in *D. melanogaster *in the process of improving deletion coverage. This information should contribute to future studies of gene dosage relationships and aneuploidy effects.

Chromosomal deletions are one of the most versatile and important genetic tools available to *Drosophila *researchers and the stocks generated in this project will contribute to the success of experimental studies for years to come. Resource development projects like ours and their targeted funding propel research by creating new experimental opportunities and they are vital to the continued vigor and advancement of scientific inquiry in genetic model organisms.

## Materials and methods

### Sources of stocks

Most *P{XP}*, *PBac{WH} *and *PBac{RB} *insertions used in deletion screens were obtained directly from Exelixis, Inc. The remainder came from the BDSC and the Exelixis collection at Harvard University. Other stocks came from Exelixis, the BDSC, the Harvard collection, the Szeged Stock Centre, the *Drosophila *Genetic Resource Center at the Kyoto Institute of Technology and the laboratory of Michael Ashburner at the University of Cambridge.

### Chromosomal positions

All genomic coordinates and gene counts are based on Genome Release 5.16. Cytological band positions were predicted from Release 5 coordinates using FlyBase map conversion tables [39,40]. We artificially defined the euchromatin/heterochromatin boundary as the most proximal extent of the contiguous genomic assembly for each chromosome arm in Genome Release 5.

The genomic coordinates of *P{XP}*, *PBac{WH} *and *PBac{RB} *insertions were initially determined at Exelixis and most were published in Thibault *et al.*[10]. Coordinates of unpublished insertions were obtained directly from Exelixis. Flanking sequences were reanalyzed by Roger Hoskins and Joe Carlson of Lawrence Berkeley National Laboratory and they submitted revised coordinates for many insertions to FlyBase. We used these reanalyzed coordinates where available. Otherwise, we used coordinates from a preliminary reanalysis (R Hoskins, personal communication) or the original Exelixis coordinates in that order of preference. The origins of coordinates are recorded in Additional file 1.

### Deletion screens

Parks *et al.*[5] described methods for using *P{XP}*, *PBac{WH} *and *PBac{RB} *insertions to isolate chromosomal deletions in a standard genetic background. We followed the basic schemes developed at Exelixis for deletion screening, but we introduced some alternative stocks to improve the vigor of flies in the crosses and final deletion stocks. We built these stocks by carefully introgressing balancer and marker chromosomes into the standard background using chromosome substitution schemes patterned on Craymer [41]. The source of the *Y *and fourth chromosomes was not controlled. (Details of stock constructions will be provided upon request.) Here we present the crosses we eventually found to work best for the isolation of *X*, second and third chromosome deletions. We identified deletions based on loss of *miniwhite *or the presence of two copies of *miniwhite*; see Park *et al.*[5] and Results for the rationale. *Tn{w^+^}1 *and *Tn{w^+^}2 *are used generically to denote different *miniwhite*-marked transposon (*Tn*) insertions of *P{XP}*, *PBac{WH} *and *PBac{RB}*. Deletions were named with a *BSC *(Bloomington Stock Center) prefix followed by a number in the order they were placed into public distribution.

#### *X *chromosome deletions

G0: *w^1118 ^Tn{w^+^}1 *♀♀ × *w^1118^/Y; MKRS, P{hsFLP}86E/TM6B, Tb^1 ^*♂♂ (9 vials of 8 ♀♀ × 5 ♂♂, subcultured once).

G1: *w^1118 ^Tn{w^+^}2 *♀♀ × *w^1118 ^Tn{w^+^}1/Y; +/MKRS, P{hsFLP}86E *♂♂ (9 vials of 8 ♀♀ × 5 ♂♂, subcultured once).

G2: *w^1118 ^Tn{w^+^}2/w^1118 ^Tn{w^+^}1; +/MKRS, P{hsFLP}86E *♀♀ × *FM7h/Dp(2;Y)G, P{hs-hid}Y *♂♂ (9 vials of 8 ♀♀ × 5 ♂♂, subcultured once; these females were heat shocked as larvae at 37°C for 1 hour on days 4 to 8 after egg lay).

G3: *Df(1)BSC, w^1118^/FM7h *♀ × *FM7h/Dp(2;Y)G, P{hs-hid}Y *♂♂ (deletion-bearing females were recognized as white-eyed or darker-eyed flies, depending on the screen; we typically established stocks of five independent putative deletions for testing).

Although the temperature-sensitive lethal *P{hs-hid} *construct can be used to eliminate unwanted progeny classes, it was incidental to our choice of stocks in the preceding and succeeding crosses and not used. We include it here only for completeness.

#### Second chromosome deletions {3rd level heading}

G0: *P{hsFLP}1, y^1 ^w^1118^; P{hs-hid}2, wg^Sp-1^/CyO *♀♀ × *w^1118^; Tn{w^+^}1 *♂♂ (5 vials of 8 ♀♀ × 5 ♂♂, subcultured once).

G1: *P{hsFLP}1, y^1 ^w^1118^/w^1118^; CyO/Tn{w^+^}1 *♀♀ × *w^1118 ^/Y; Tn{w^+^}2 *♂♂ (5 vials of 8 ♀♀ × 5 ♂♂, subcultured once).

G2: *w^1118^; wg^Sp-1^/SM6a *♀♀ × *P{hsFLP}1, y^1 ^w^1118^/Y; Tn{w^+^}1/Tn{w^+^}2 *♂♂ (5 vials of 8 ♀♀ × 5 ♂♂, subcultured once; these males were heat shocked as larvae at 37°C for 1 hour on days 4 to 8 after egg lay).

G3: *w^1118^; wg^Sp-1^/SM6a *♀♀ × *w^1118^/Y; Df(2)BSC/SM6a *♂ (deletion-bearing males were recognized as white-eyed or darker-eyed flies, depending on the screen; we typically established stocks of five independent putative deletions for testing.)

G4: *w^1118^; Df(2)BSC/SM6a *♀♀ × *w^1118^; Df(2)BSC/SM6a *♂♂.

#### Third chromosome deletions {3rd level heading}

G0: *P{hsFLP}1, y^1 ^w^1118^; Dr^1^/TM3, Sb^1 ^*♀♀ × *w^1118^; Tn{w^+^}1 *♂♂ (5 vials of 8 ♀♀ × 5 ♂♂, subcultured once).

G1: *P{hsFLP}1, y^1 ^w^1118^/w^1118^; TM3, Sb^1^/Tn{w^+^}1 *♀♀ × *w^1118^/Y; Tn{w^+^}2 *♂♂ (5 vials of 8 ♀♀ × 5 ♂♂, subcultured once).

G2: *w^1118^; P{hs-hid}3, Dr^1^/TM6C, cu^1 ^Sb^1 ^*♀♀ × *P{hsFLP}1, y^1 ^w^1118^/Y; Tn{w^+^}1/Tn{w^+^}2 *♂♂

(5 vials of 8 ♀♀ × 5 ♂♂, subcultured once; these males were heat shocked as larvae at 37°C for 1 hour on days 4 to 8 after egg lay).

G3: *w^1118^; P{hs-hid}3, Dr^1^/TM6C, cu^1 ^Sb^1 ^*♀♀ × *w^1118^/Y; Df(3)BSC/TM6C, cu^1 ^Sb^1 ^*♂ (deletion-bearing males were recognized as white-eyed or darker-eyed flies, depending on the screen; we typically established stocks of five independent putative deletions for testing).

G4: *w^1118^; Df(3)BSC/TM6C, cu^1 ^Sb^1 ^*♀♀ × *w^1118^; Df(3)BSC/TM6C, cu^1 ^Sb^1 ^*♂♂.

It was necessary to establish stocks of a few deletions in nonstandardized backgrounds to overcome problems arising from noncomplementation or haploinsufficiency, but these stocks are noted in Additional file 1 and BDSC stock records.

### Verifying new deletions

Four approaches were used to verify that the planned deletion was recovered from a screen: complementation tests, polytene chromosome cytology, 'hybrid PCR' and PCR with primers flanking transposable element insertions. The complementation tests were routine and require no elaboration. Polytene chromosomes were analyzed in standard lacto-aceto-orcein preparations [42] using the maps of Lefevre [43] and Sorsa *et al.*[44,45].

The 'hybrid PCR' approach was described in Parks *et al.*[5]. Briefly, primers were used to amplify a DNA fragment from the recombinant transposable element formed upon FLP-mediated recombination between two progenitor *FRT*-bearing insertions. We used the primers described in Parks *et al.*[5] with the substitution of a new primer (5'-GCTTCTAAACGCTTACGCATAAACGATG-3') for the RB3' plus and RB3' minus primer. We were unable to establish Hybrid PCR primers and conditions to verify deletions identified from an increase in *miniwhite *copy number; consequently, we tested only deletions detected by loss of *miniwhite *markers.

To verify that a particular chromosomal region was removed by a deletion, we designed PCR primers flanking the insertion site of a transposon located within the deleted region. From crosses, we isolated progeny carrying both the transposon and the putative deficiency and isolated DNA. With short extension times, a PCR fragment is amplified only when there is no transposon between the primer sites; consequently, a fragment is recovered if the noninsertion chromosome lacks a deletion. Since this approach verifies a deletion by a negative result, we repeated the test with three to five independent DNA preparations and appropriate positive controls. Transposon insertions and primer sequences used in these tests are given in Additional file 1. Additional details are available from Cook *et al.*[21] where similar tests were used to confirm chromosomal duplications.

Stocks were placed into public distribution by the BDSC as soon as we verified deletions. We submitted a description of each deletion to FlyBase and Additional file 1 provides citations to these reports.

### Stock availability

Stocks described in this paper may be obtained from the BDSC. Information on ordering, web pages devoted to deletion collections and the Deficiency Kit and lists of other stocks are available on the BDSC website [46]. FlyBase [39] provides information on individual genetic components with cross references to BDSC stocks.

## Abbreviations

BDSC: Bloomington *Drosophila *Stock Center; FLP: FLP recombinase; *FRT*: FLP recognition target; PCR: polymerase chain reaction; SNP: single-nucleotide polymorphism.

## Competing interests

The authors declare that they have no competing interests.

## Authors' contributions

RKC, SJC, JAD, RAC, MED and JMG planned and conducted genetic screens and characterized new deletions. KRC initiated and supervised the project, characterized deletions, analyzed data, prepared stocks for public distribution and wrote the manuscript. RKC analyzed data and revised the manuscript. TCK provided support and guidance. All authors read and approved the final manuscript.

## Supplementary Material

Additional file 1 **Breakpoints and characterization of BSC deletions**. This file provides full information for deletions isolated in this project, including progenitor *FRT *insertions and their locations, selection criteria in screens, verification tests and references to FlyBase documentation. Click here for file

Additional file 2 **Map of *X *and fourth chromosome deletion breakpoints**. This spreadsheet shows the breakpoints and progenitor FRT insertions of Exelixis, DrosDel and BSC deletions positioned relative to the proximal and distal ends of annotated genes. It also shows the breakpoints of other deletions in the BDSC Deficiency Kit. The filled-in cells in columns to the right depict deletions graphically. Similarly, the positions of haploinsufficient genes are highlighted in colored rows to show their relationships to the deletions. Notes explain how we estimated the positions of breakpoints for deletions in the Deficiency Kit that have not been molecularly characterized. Click here for file

Additional file 3 **Haploinsufficient genes with viability and fertility effects**. This table gives details of the mapping and identification of haplolethal and haplosterile genes in *D. melanogaster*, including cytological locations, hemizygous phenotypes, deletions removing the genes, deletions most closely flanking the genes and the number of closely linked genes sharing the same deletion breakpoint interval. Click here for file

Additional file 4 **Bloomington *Drosophila *Stock Center Deficiency Kit stocks**. This file lists the component deletions in the BDSC Deficiency Kit with their breakpoints and stock information. Click here for file
